# Complete-genome sequencing and comparative genomic characterization of *bla*_NDM-5_ carrying *Citrobacter freundii* isolates from a patient with multiple infections

**DOI:** 10.1186/s12864-023-09579-9

**Published:** 2023-08-30

**Authors:** Jianzhong Ye, Lulu Jin, Yaling Li, Hao Xu, Yishuai Lin, Tieli Zhou, Beiwen Zheng, Maofeng Wang, Zhongyong Wang

**Affiliations:** 1 Department of Clinical Laboratory, Key Laboratory of Clinical Laboratory Diagnosis and Translational Research of Zhejiang Province, the First Affiliated Hospital of Wenzhou Medical University, Nanbaixiang Street, Ouhai District, Wenzhou, 325000 Zhejiang Province China; 2 Department of Biomedical Sciences Laboratory, Affiliated Dongyang Hospital of Wenzhou Medical University, No. 60 Wuning West Road, Dongyang, 322100 Zhejiang Province China; 3 Department of Health Screening Center, The Second Affiliated Hospital, School of Medicine, Zhejiang University, Hangzhou, 310000 Zhejiang China; 4 Wenzhou Medical University, Wenzhou, 325000 Zhejiang China; 5 State Key Laboratory for Diagnosis and Treatment of Infectious DiseasesCollaborative Innovation Center for Diagnosis and Treatment of Infectious DiseasesSchool of Medicine, National Clinical Research Center for Infectious Diseasesthe First Affiliated HospitalZhejiang University, No. 79 Qingchun Road, Shangcheng District, Hangzhou, 310000 Zhejiang Province China

**Keywords:** Antimicrobial resistance, Horizontal gene transfer, IncX plasmid, Plasmid conjugation

## Abstract

**Background:**

The emergence and wide spread of carbapenemase-producing *Enterobacteriaceae* (CPE) poses a growing threat to global public health. However, clinically derived carbapenemase-producing *Citrobacter* causing multiple infections has rarely been investigated. Here we first report the isolation and comparative genomics of two *bla*_NDM-5_ carrying *Citrobacter freundii* (*C. freundii*) isolates from a patient with bloodstream and urinary tract infections.

**Results:**

Antimicrobial susceptibility testing showed that both *bla*_NDM-5_ carrying *C. freundii* isolates were multidrug-resistant. Positive modified carbapenem inactivation method (mCIM) and EDTA-carbapenem inactivation method (eCIM) results suggested metallo-carbapenemase production. PCR and sequencing confirmed that both metallo-carbapenemase producers were *bla*_NDM-5_ positive. Genotyping and comparative genomics analyses revealed that both isolates exhibited a high level of genetic similarity. Plasmid analysis confirmed that the *bla*_NDM-5_ resistance gene is located on IncX3 plasmid with a length of 46,161 bp, and could successfully be transferred to the recipient *Escherichia coli* EC600 strain. A conserved structure sequence (IS*Aba125*-IS*5*-*bla*_NDM-5_-*trpF*-IS*26*-*umuD*-IS*Kox3*) was found in the upstream and downstream of the *bla*_NDM-5_ gene.

**Conclusions:**

The data presented in this study showed that the conjugative *bla*_NDM-5_ plasmid possesses a certain ability to horizontal transfer. The dissemination of NDM-5-producing *C. freundii* isolates should be of close concern in future clinical surveillance. To our knowledge, this is the first study to characterize *C. freundii* strains carrying the *bla*_NDM-5_ gene from one single patient with multiple infections.

**Supplementary Information:**

The online version contains supplementary material available at 10.1186/s12864-023-09579-9.

## Background

The emergence and wide spread of carbapenem-resistant *Enterobacteriaceae* (CRE) has become a major threat to global public health [1]. Resistant mechanism of CRE to carbapenems is mainly due to the production of carbapenemases, which are enzymes able to recognize almost all hydrolyzable β-lactams, including carbapenems [2]. New Delhi metallo-β-lactamase (NDM) is one of the main types of carbapenemases, and are resilient against inhibition by commercially available β-lactamase inhibitors, including avibactam, clavulanate, sulbactam, and tazobactam [3]. Since the first detection of *bla*_NDM-1_, 48 variants of NDM enzymes (NDM-1 to NDM-16a, 16b, and NMD-17 to NDM-48, except NDM-32) have been identified worldwide (https://www.ncbi.nlm.nih.gov/pathogens/refgene/#NDM, accessed 29 December 2022). Among them, NDM-5 has raised extensive concerns for increased resistance to carbapenems and expanded-spectrum cephalosporins, since first determined in a multidrug-resistant *Escherichia coli* ST648 isolate in the United Kingdom in 2011 [4].

*Citrobacter freundii*, belonging to the genus *Citrobacter* of the family *Enterobacteriaceae*, is rarely the causative pathogen of infections but can cause a wide spectrum of opportunistic nosocomial infections including respiratory tract, urinary tract, and bloodstream [5]. Additionally, it can lead to neonatal meningitis and brain abscesses, which are associated with high mortality rates [6]. The emergence of multidrug resistant *C. freundii* strains, particularly those that produce carbapenemase enzymes, has posed challenges for infection treatment and has become an increasing global public health concern. This is especially true for immunocompromised patients, who heavily rely on antibiotics [7].

In this study, we presented the results of complete-genome sequencing and comparative genomic characterization of two NDM-5 producing *C. freundii* strains isolated from a patient with concurrent bloodstream and urinary tract infections.

## Results

### Antimicrobial resistance profiles of both *C. freundii* isolates

Antimicrobial susceptibility testing of both *C. freundii* isolates revealed high MIC values for different drugs and different susceptibility-resistance levels depending on the drug tested. However, both isolates exhibited similar phenotypic antibiotic susceptibility, demonstrating a multi-drug resistant (MDR) characteristic. Both *C. freundii* isolates exhibited resistance to amoxicillin/clavulanic acid, piperacillin/tazobactam, ceftazidime, ceftriaxone, cefepime, cefotaxime, ciprofloxacin, levofloxacin, trimethoprim/sulfamethoxazole, and gentamicin as shown in Table 1. In contrast, isolates remained susceptible to amikacin, aztreonam, fosfomycin and tigecycline (Table 1). Intermediate resistance was exhibited when isolates were cultured in the presence of imipenem, meropenem and polymixin B (Table 1).

**Table 1:** Antimicrobial susceptibility profiles of the two isolates and corresponding transconjugant

	**MIC (mg/L)/Antimicrobial susceptibility**			
**Antibiotic**	DY2007	DY2007-NDM-EC600	DY2010	DY2010-NDM-EC600
Amoxicillin/Clavulanic acid	128/R	128/R	128/R	128/R
Piperacillin/Tazobactam	> 128/R	> 128/R	> 128/R	> 128/R
Ceftazidime	> 128/R	> 128/R	> 128/R	> 128/R
Ceftriaxone	> 128/R	> 128/R	> 128/R	> 128/R
Cefepime	32/R	32/R	32/R	32/R
Cefotaxime	> 128/R	> 128/R	> 128/R	> 128/R
Ciprofloxacin	> 64/R	0.5/S	> 64/R	0.5/S
Levofloxacin	32/R	1/I	64/R	1/I
Imipenem	2/I	2/I	2/I	2/I
Meropenem	2/I	2/I	2/I	2/I
Trimethoprim/Sulfamethoxazole	> 152/R	< = 2.375/S	> 152/R	< = 2.375/S
Amikacin	4/S	4/S	4/S	4/S
Gentamicin	> 128/R	2/S	> 128/R	2/S
Aztreonam	0.5/S	0.25/S	1/S	0.25/S
Fosfomycin	64/S	8/S	64/S	8/S
Tigecycline	0.25/S	< 0.05/S	1/S	< 0.05/S
Polymixin B	1/I	0.5/I	2/I	0.5/I
Tetracycline	> 128/R	2/S	> 128/R	1/S
Azithromycin	64/R	16/S	64/R	16/S
Rifamycin	> 128/NA	> 128/ NA	> 128/ NA	> 128/ NA

*S* susceptible, *R* resistant, *I* intermediate, *NA* not applicable

### Phenotype and genotype detection revealed the mechanism of carbapenems non-susceptibility

Positive modified carbapenem inactivation method (mCIM) and EDTA-carbapenem inactivation method (eCIM) results suggested metallo-carbapenemase production of both *C. freundii* isolates. Then, PCR and sequencing were used to study the molecular determinant, and the results demonstrated that both metallo-carbapenemase producers were *bla*_NDM-5_ positive, thus confirming their intermediate resistance phenotype towards imipenem and meropenem. Furthermore, in silico analysis showed that both *bla*_NDM-5_ carrying isolates carried additional resistance genes conferring resistance to β-lactams (*bla*_TEM-1B_, *bla*_OXA-1_, *bla*_CMY-48_, *bla*_DHA-1_), this genetic profile correlated with their phenotypic resistance to ceftazidime, ceftriaxone, cefepime, and cefotaxime. Moreover, they carried resistance genes for aminoglycosides (*aac(6')-Ib-cr*, *aac(3)-IId*, *aadA1*), which correlated with phenotypic resistance to gentamicin. The presence of the *qnrB4* gene indicated resistance to quinolones, which was in line with their resistance to ciprofloxacin and levofloxacin. Furthermore, the isolates carried resistance genes for trimethoprim/sulfamethoxazole (*dfrA1*, *sul1*, *sul2*), which were associated with their resistance to trimethoprim/sulfamethoxazole. The presence of amphenicol resistance genes (*catA2*, *catB3*), a tetracycline resistance gene *tet(D)*, and a macrolides resistance gene *mph(A)* correlated with their resistance to ciprofloxacin, tetracycline, and azithromycin, respectively. Although the presence of the rifamycin resistance gene *ARR-3* contributed to both isolates having MIC values exceeding 128 mg/L for rifamycin, the absence of a defined breakpoint prevented the determination of the resistance phenotype with certainty.

### High degree of genetic similarity of both NDM-5-producing *C. freundii* isolates

**Fig. 1:**
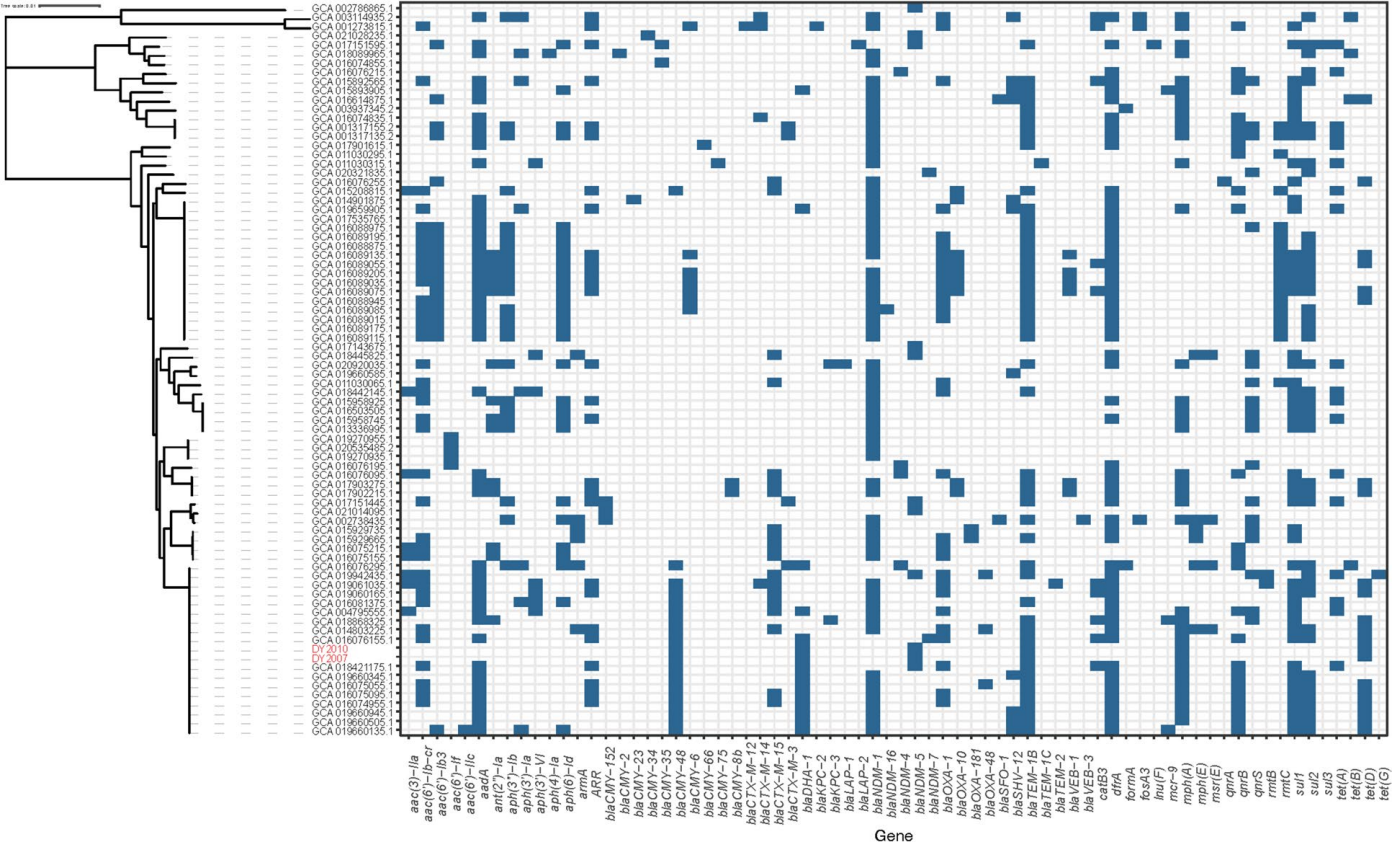
The core-genome phylogenetic tree generated by kSNP. Blue color of the right panel indicates positive antibiotic resistance genes of the corresponding strains

An average nucleotide identity blast (ANIb) analysis, which measures the nucleotide-level genomic similarity between the coding regions of two genomes, was performed. The analysis revealed that the two isolates exhibited a remarkable similarity of over 99% (Supplementary Fig. 1). Additionally, when core single nucleotide polymorphisms (SNPs) were calculated, only five base differences were detected between the two isolates. MLST typing revealed that both NDM-5-producing *C. freundii* isolates belonged to the sequence typing ST 22. Single-nucleotide polymorphism (SNP)-based phylogenetic tree for both isolates and other 78 NDM-producing strains indicated that DY2007 and DY2010 showed high degree of similarity, and were closely related to various strains from different countries, including Myanmar, USA, France, Australia, Malaysia, Germany, China, and Singapore (Fig. 1, Table S1).

### Characterization of both *bla*_NDM-5_ carrying plasmids

**Fig. 2:**
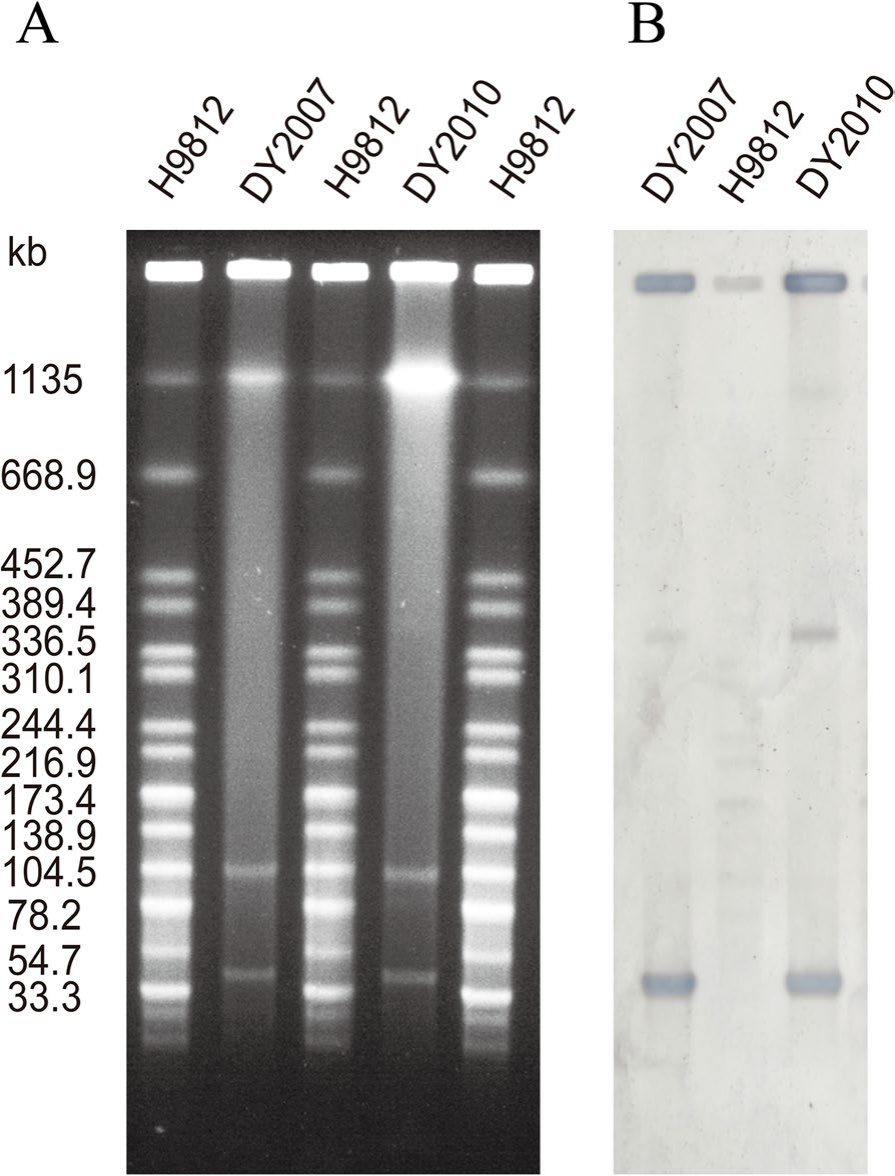
*bla*_NDM-5_ gene location analysis. **A** S1-PFGE of both *bla*_NDM-5_ carrying *C. freundii* isolates DY2007 and DY2010. *Salmonella enterica* serotype H9812 was used as molecular marker. **B** Corresponding Southern blotting analysis using *bla*_NDM-5_-specific probe. A and B was cropped from different gels. Full-length blots/gels are presented in Supplementary Fig. 2

S1-PFGE and Southern blotting revealed that both *C. freundii* isolates contained a ~ 50 kb plasmid harbouring the *bla*_NDM-5_ gene (Fig. 2). Plasmid replicons analysis indicated that both pNDM-5 were IncX3 type with a length of 46,161 bp. The results of the conjugation assay showed the *bla*_NDM-5_ gene of both isolates could successfully be transferred to the recipient *Escherichia coli* EC600 strain. The results were further confirmed by PCR using *bla*_NDM-5_ specific primers and sequencing.

### Whole-genome sequencing analysis of both *bla*_NDM-5_ carrying *C. freundii* isolates

**Fig. 3:**
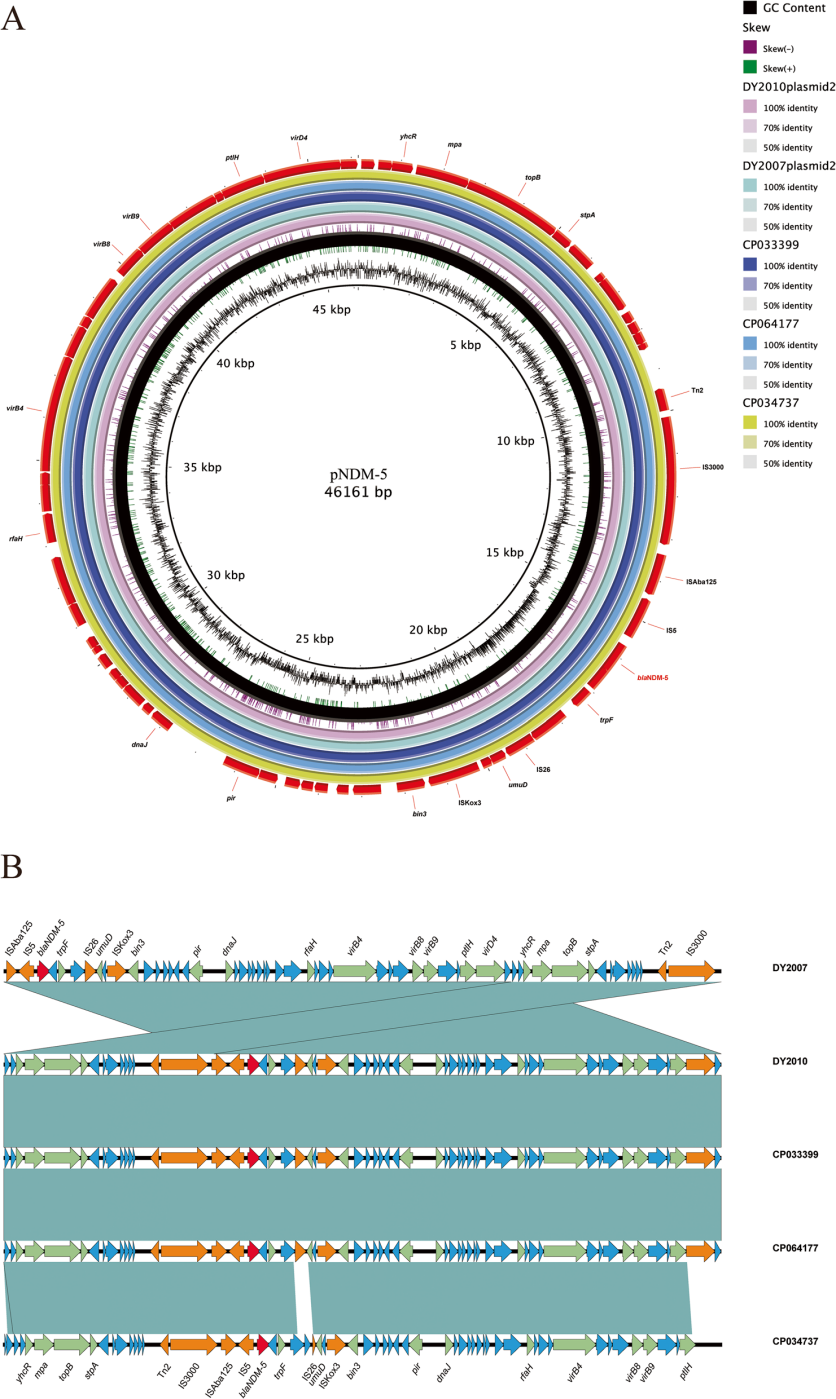
Genomic analyses of pNDM-5 plasmid. **A** Comparison of the pNDM-5 plasmid sequence identified in isolates DY2007 and DY2010 with *Escherichia coli* strain WCHEC020031 plasmid pNDM5_020031 (GenBank accession number: CP033399.1), *Klebsiella pneumoniae* strain 19,110,124 plasmid p19110124-3 (GenBank accession number: CP064177.1), and *Escherichia coli* strain L53 plasmid pL53-4 (GenBank accession number: CP034737.1). The figure was plotted using BRIG, and the *bla*_NDM-5_ gene was highlighted in red. **B** Genetic environment of *bla*_NDM-5_ on pNDM-5 and related plasmids. Open reading frames were indicated as arrows. Shared areas with highly similar sequences were drawn by lake green. Conjugal transfer associated genes were shown by brown; *bla*_NDM-5_ gene were indicated by red arrows; functional protein coding genes were colored by green; other antibiotic resistance genes were colored by blue

The genomic characteristics of both *bla*_NDM-5_ carrying *C. freundii* isolates were shown in Table S2. DY2007 contained a circular chromosome and two plasmids with a genome size of 5,253,532 bp, while DY2010 consisted of a circular chromosome and three plasmids with a genome size of 5,260,876 bp. The average GC content of both genomes was 51.6%. The complete sequence of *bla*_NDM-5_ carrying pNDM-5 was covered using combinatorial PCR and standard Sanger sequencing to accomplish sequence integrality of contigs. It was found that both isolates harbored an identical pNDM-5 plasmid of 46,161 bp length, with a GC content of 46.65% and 65 predicted coding sequences (Fig. 3A). The plasmid carried multiple coding genes, including IncX plasmid conjugal transfer associated genes, antibiotic resistance genes, stability related genes, functional protein coding genes, mobile element associated genes, and other hypothetical genes. A search of the nr/nt database revealed a 100% identity to *Escherichia coli* strain WCHEC020031 plasmid pNDM5_020031 (GenBank accession number: CP033399.1) at 100% coverage, 100% identity to *Klebsiella pneumoniae* strain 19110124 plasmid p19110124-3 (GenBank accession number: CP064177.1) at 100% coverage, and 99.98% identity to *Escherichia coli* strain L53 plasmid pL53-4 (GenBank accession number: CP034737.1) at 99% coverage. Furthermore, a conserved structure sequence (IS*Aba125*-IS*5*-*bla*_NDM-5_-*trpF*-IS*26*-*umuD*-IS*Kox3*) was found in the upstream and downstream of *bla*_NDM-5_ (Fig. 3B).

## Discussion

Pathogenic carbapenemase-producing *Citrobacter freundii* has been individually isolated from bloodstream, feces, open pus, and urine samples [6–9]. Carbapenem-resistant genes frequently found in *C. freundii* were *bla*_KPC_, *bla*_NDM_, *bla*_VIM_, *bla*_OXA-48_, and *bla*_IMP_ [5–7, 10]. In this study, we reported the complete-genome sequencing and comparative genomic characterization of two *bla*_NDM-5_ carrying *C. freundii* isolates from an inpatient with concurrent bloodstream and urinary tract infection. To the best of our knowledge, this is the first study to characterize *C. freundii* strains carrying the *bla*_NDM-5_ gene from one single patient with multiple infections.

Antibiotic susceptibility testing indicated the multidrug-resistant characteristic of both isolates, with resistant to classical β-lactams, aminoglycosides, macrolides, quinolones, and sulfonamides, and intermediate to polypeptide antibiotics and carbapenems, the resistant phenotype of which was consistent with resistant genotype (Table 1).

Phylogenetic analysis determined the high homology of both ST 22 isolates, which further highlighted the potential threat of systemic infections caused by this multidrug-resistant (MDR) strain. The multidrug-resistant *bla*_NDM-5_ carrying *C. freundii* isolates have been reported previously from South Korea and Nigeria [8, 9].

The New Delhi metallo-beta-lactamase NDM-5 was first reported in a multidrug-resistant *Escherichia coli* ST648 isolate, recovered from a patient in the United Kingdom [4]. Substitutions at positions 88 (Val → Leu) and 154 (Met → Leu) distinguished NDM-5 from NDM-1 and enhanced its hydrolytic activity toward carbapenems [11]. Since then, dissemination of NDM-5-producing *Enterobacteriaceae* has been reported worldwide in medical units and environment [12, 13]. Except for *C. freundii*, the emergence of the *bla*_NDM-5_ gene had been reported in *Escherichia coli*, *Klebsiella pneumoniae*, *Klebsiella aerogenes*, *Klebsiella oxytoca*, *Proteus mirabilis*, and *Morganella morganii* [12, 14–18].

Compared to vertical transmission, horizontal transfer seems to be more threatening for antimicrobial resistance. In this study, two isolates both successfully transferred *bla*_NDM-5_ and carbapenem non-susceptible phenotype to the recipient strain *E. coli* EC600, which confirmed the horizontal gene transfer characteristic of NDM-5. In recent years, IncX3 plasmids harboring *bla*_NDM_ variants have increasingly been characterized worldwide. It has also been proved that IncX3 plasmids plays an important role in the dissemination of *bla*_NDM-5_ gene in *Enterobacteriaceae* [12]. Tian et al. observed that horizontal gene transfer (HGT) of *bla*_NDM-5_ among distinct *Enterobacteriaceae* species was mostly mediated by IncX3 plasmids [16]. A previous study found that *bla*_NDM-5_ might spread among humans and the environment via IncX3 plasmids in an intensive vegetable farming area in eastern China [19]. Consistently, *bla*_NDM-5_ was located on an IncX3 plasmid (~ 50 kb) in this study. However, *bla*_NDM-5_ was additionally detected on IncF and IncI1 plasmids [20, 21], highlighting the significant compatibility and transmission hazard associated with *bla*_NDM-5_.

In order to further understand the evolutionary relationship of *bla*_NDM-5_ harboring *C. freundii* isolates, we downloaded the nucleotide sequences of 78 NDM-producing strains from NCBI and analyzed their homology with both isolates in this study. The results revealed that 17 strains from around the world were closely related to both isolates, suggesting parallel evolution of these isolates. In plasmids, genes are typically associated with mobile genetic elements such as transposons (Tn) and insertion sequences (IS). According to the whole-genome sequencing analysis of the pNDM-5 plasmids, the comparison of genetic context flanking *bla*_NDM-5_ in both isolates was mostly identical to published plasmids, i.e., IS*Aba125*-IS*5*-*bla*_NDM-5_-*trpF*-IS*26*-*umuD*-IS*Kox3*. The *bla*_NDM-5_ genetic structure is widespread in *Enterobacteriaceae* for *bla*_NDM_ horizontal transfer and has been reported in *bla*_NDM-5_ and *bla*_NDM-9_ transmission [22]. Characterization of the *bla*_NDM-5_ genetic contents revealed that it was flanked by multi-insertional sequences. Of these, IS*Aba125* was conservative in *bla*_NDM-5_-positive isolates. It is consistent with the discovery that IS*Aba125* (intact or truncated) upstream of *bla*_NDM_ is common in *bla*_NDM_ genetic settings [23], indicating its’ important role in *bla*_NDM_ transmission. NDM, a highly prevalent plasmid-borne metallo-β-lactamase, has been identified in various species of *Enterobacteriaceae* worldwide. Its frequent co-occurrence with IS*Aba125* suggests a potential origin from *Acinetobacter* spp., a bacterium where this association is commonly observed [24]. Tn125, a composite transposon based on IS*Aba125*, has been reported as one of the genetic elements implicated in the dissemination of *bla*_NDM_. However, in *Enterobacteriaceae*, Tn125 exhibits interruptions or truncations, leading to diverse genetic contexts for *bla*_NDM_ [3].

Whole-genome sequencing (WGS) plays a pivotal role in clinical cases involving systemic infections. It offers a comprehensive perspective on the complete genome of the pathogen, allowing for meticulous analysis of genetic variations, resistance mechanisms, and the identification of potential virulence factors. WGS enhances our understanding of the pathogenesis of systemic infections, aids in tracing transmission routes, and assists in formulating suitable treatment strategies [25–27]. This study bears significance in the ongoing efforts to incorporate WGS into routine clinical diagnostic pipelines.

## Conclusions

In this study, we sequenced and characterized the comparative genomes of two *bla*_NDM-5_ carrying *C. freundii* isolates from an inpatient with multiple infections, and found different antimicrobial resistant genes in a transferable IncX3-type plasmid. The dissemination of this MDR isolate should be of close concern in future clinical surveillance.

## Materials and methods

### Case presentation and bacterial isolates

Two *C. freundii* isolates were collected from a 62 years old female bladder cancer inpatient with multiple infections of the Affiliated Dongyang Hospital of Wenzhou Medical University (Wenzhou, China) in 2020. One isolate (DY2007) was first recovered from bloodstream on January 21 and the other one (DY2010) was subsequently obtained from urinary tract on February 12. The patient presented with lower back pain, accompanied by fever and chills, and was admitted on January 20, 2020. She received intravenous administration of 2g of ceftriaxone-sulbactam every 8 h for antimicrobial treatment until February 1. Additionally, on January 23, based on the strain's antimicrobial susceptibility testing results, amikacin injection was added to the treatment regimen at a dosage of 0.2g every 12 h. This treatment continued until February 1, when the patient's symptoms improved, and she requested discharge. Subsequently, on February 9, the patient was readmitted due to lower back pain and received the aforementioned amikacin treatment until February 17, when the patient made a complete recovery and was subsequently discharged. The strains were isolated using sheep blood agar cultured overnight at 37 ^◦^C and were initially identified using MALDI-TOF MS (BioMérieux, France). The isolates were stored in 30% glycerol at -80 ^◦^C until further analysis.

### Antimicrobial susceptibility testing

The minimum inhibitory concentrations (MICs) of 17 antibiotics, including amoxicillin/clavulanic acid, piperacillin/tazobactam, ceftazidime, ceftriaxone, cefepime, cefotaxime, ciprofloxacin, levofloxacin, imipenem, meropenem, trimethoprim/sulfamethoxazole, amikacin, gentamicin, aztreonam, fosfomycin, tigecycline, and polymixin B, were determined by the VITEK 2 system with AST-GN13 card and the agar dilution method. The breakpoint of tigecycline was interpreted according to the recommendations of the Food and Drug Administration (FDA) [28], and the breakpoint of other antibiotics were interpreted according to the Clinical and Laboratory Standards Institute (CLSI) 2021 guidelines [29].

### Carbapenemases detection and molecular mechanisms identification

The mCIM and eCIM method were used to determine carbapenemase production according to the CLSI guidelines. Carbapenemase genes (*bla*_KPC_, *bla*_NDM_, *bla*_IMP_, *bla*_VIM_, and *bla*_OXA-48_) were identified by PCR amplification as in our previous publication [30]. Positive amplification products were then sequenced for verification and subtype typing.

### Sequence typing and genetic relationship analyses

Multilocus sequence typing (MLST) analysis of both *C. freundii* isolates was undertaken by amplifying seven housekeeping genes (*aspC*, *clpX*, *fadD*, *mdh*, *arcA*, *dnaG*, and *lysP*). The sequence type was assigned by allelic profile comparison using the pubMLST database (https://pubmlst.org/cfreundii/). A single-nucleotide polymorphism (SNP)-based phylogenetic tree, including the genome of both *C. freundii* isolates sequenced in this study and 78 additional genomes of NDM-producing strains downloaded from the NCBI GenBank database (Table S1) [31], was constructed. To do so, a core SNPs matrix was calculated by comparing such genomes using the kSNP version 3 (https://sourceforge.net/projects/ksnp/files/) [32] and used to generate a maximum likelihood tree using iTOL version 5 (https://itol.embl.de/) [33].

### Plasmid characterization and conjugation assay

Plasmid sizes of the strains were determined using the S1 nuclease pulsed-field gel electrophoresis (S1-PFGE) method. The location of the *bla*_NDM-5_ gene was investigated by Southern blotting with a specific digoxigenin-labelled *bla*_NDM-5_ probe using the DIGHigh Prime DNA Labeling and Detection Starter Kit II (Roche Diagnostics, Germany) [10]. Replicon types of plasmid incompatibility (Inc) groups were identified by multiplex PCR as previously described [10]. The plasmid conjugation and transformation methods were performed to verify the transferability of the NDM-bearing plasmid with *E. coli* 600 as a recipient strain. The transconjugants were then screened on BHI agar plates supplemented with 2 mg/L meropenem and were identified by MALDI-TOF MS. NDM-bearing recipient strain was confirmed by PCR and sequencing.

### Whole genome sequencing

Genomic DNA of both NDM-5 producing *C. freundii* isolates was extracted using the QIAmp DNA Mini Kit (Qiagen, Germany). A Qubit Fluorometer (Thermo scientific, USA) was then used to determine the concentration and purity of DNA. Sequencing libraries were prepared using the Illumina Nextera XT Kit and sequenced using Illumina HiSeq 4000-PE150 platform (Illumina, USA). Raw sequencing data of both isolates were assembled using SOAP de novo software [34] and were deposited in GenBank under the following accession numbers: JAJDSQ000000000, and CP086287-CP086290, respectively. Antimicrobial resistance genes and plasmid replicon types were matched to the Center for Genomic Epidemiology (http://www.genomicepidemiology.org/) Resfinder and Plasmid finder databases. The gaps were covered using combinatorial PCR to accomplish sequence integrality of contigs. The RAST server (http://rast.nmpdr.org/) was used to annotate the bacterial genomes, and the ISFinder database (https://www-is.biotoul.fr/) was used to identify IS elements and transposons. Multiple plasmid alignment was conducted and plotted between the *bla*_NDM-5_-harboring plasmid (named pNDM-5) and the reference plasmid using the BLAST Ring Image Generator (BRIG) [35]. Easyfig 2.2.3 was used to analyze the genetic environment surrounding the *bla*_NDM-5_ resistance gene [36].

### Average nucleotide identity blast analysis

ANIb analysis was conducted using PyANI (https://github.com/widdowquinn/pyani). The analysis included the following genomes: strain DY2007, DY2010, and five reference strains of the *Citrobacter* genus, namely *Citrobacter koseri* ATCC BAA895, *Citrobacter rodentium* ATCC51459, *Citrobacter braakii* ATCC51113, *Citrobacter youngae* ATCC29220, and *Citrobacter freundii* ATCC8090. Pairwise ANIb data for each strain were clustered and visualized using a heatmap. All aforementioned software was utilized with their default settings.

### Supplementary Information

**Additional file 1: Supplementary Figure 1. **Heatmap and dendrogram of ANIb values of DY2007, DY2010 and 5 reference strains of genus *Citrobacter*. ANIb, average nucleotide identity blast. **Supplementary Figure 2. **Full-length gels and blots figure that was used to crop in Fig. 2.

**Additional file 2: Table S1. **Information of DY2007, DY2010 and other 78 NDM-producing* C. freundii* strains used for phylogenetic tree construction.

**Additional file 3: Table S2. **List of information for both genomes that were sequenced in this study.

## Data Availability

The datasets presented in this study can be found in NCBI repository (https://www.ncbi.nlm.nih.gov/bioproject/PRJNA766039).

## Abbreviations

CPE: Carbapenemase-producing *Enterobacteriaceae*
*C.**freundii*: *Citrobacter* *freundii*
mCIM: Modified carbapenem inactivation method
eCIM: EDTA-carbapenem inactivation method
MLST: Multilocus sequence typing
SNP: Single-nucleotide polymorphism
CRE: Carbapenem-resistant *Enterobacteriaceae*
NDM: New Delhi metallo-β-lactamase
MICs: Minimum inhibitory concentrations
FDA: Food and Drug Administration
CLSI: Clinical and Laboratory Standards Institute
S1-PFGE: S1 nuclease pulsed-field gel electrophoresis
BRIG: BLAST ring image generator
ANIb: Average nucleotide identity blast
