# Supplementary material for: Complete-genome sequencing and comparative genomic characterization of blaNDM-5 carrying Citrobacter freundii isolates from a patient with multiple infections
Source: BMC Genomics. 2023 Aug 30;24:506. doi: 10.1186/s12864-023-09579-9 (PMC10466682; doi:10.1186/s12864-023-09579-9)
Supplement: Supplementary file 1 — Additional file 1: Supplementary Figure 1. Heatmap and dendrogram of ANIb values of DY2007, DY2010 and 5 reference strains of genus Citrobacter. ANIb, average nucleotide identity blast. Supplementary Figure 2. Full-length gels and blots figure that was used to crop in Fig. 2. [file 12864_2023_9579_MOESM1_ESM.docx]

**Supplementary Figure 1** Heatmap and dendrogram of ANIb values of DY2007, DY2010 and 5 reference strains of genus *Citrobacter*. ANIb, average nucleotide identity blast.


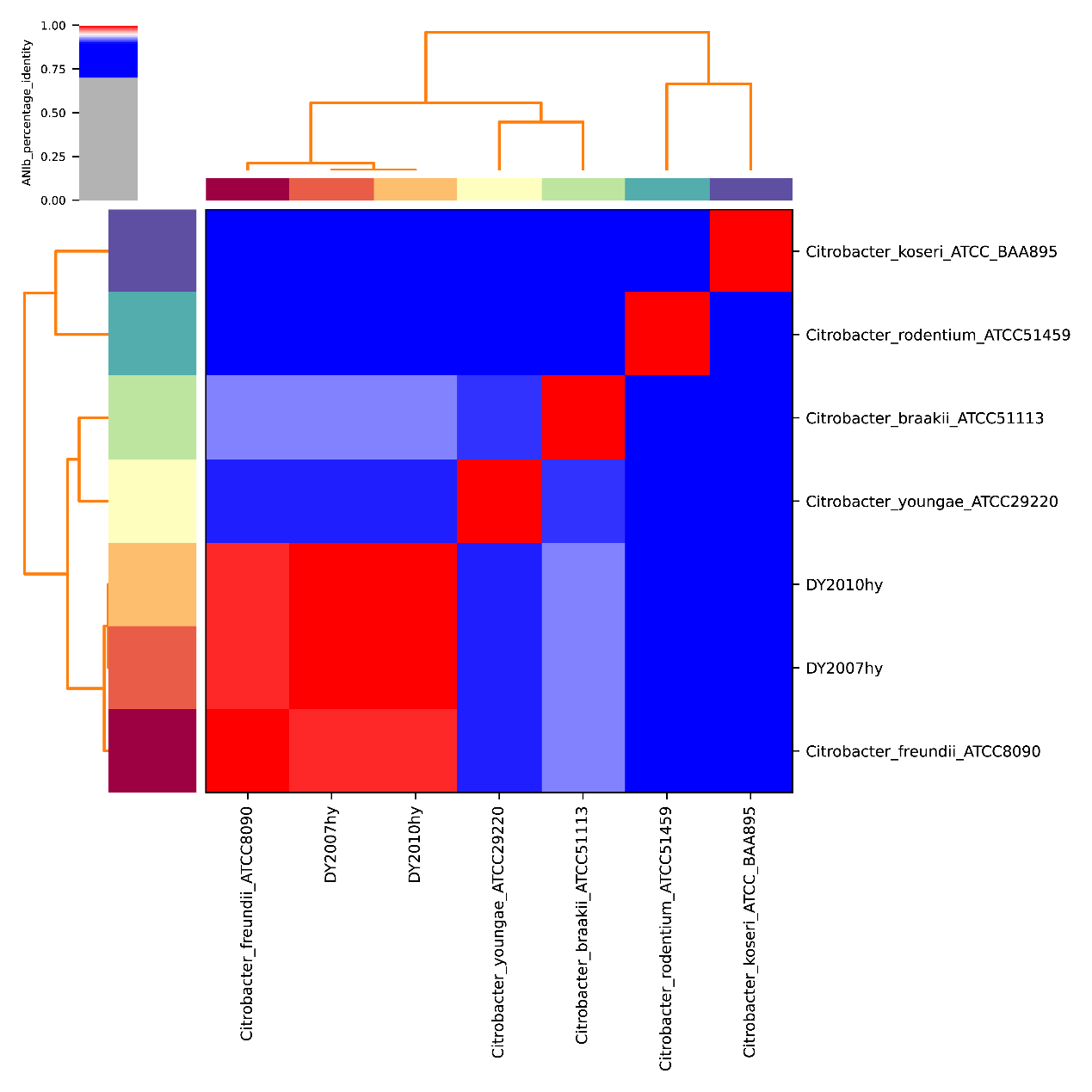


**Supplementary Figure 2** Full-length gels and blots figure that was used to crop in Fig. 2.


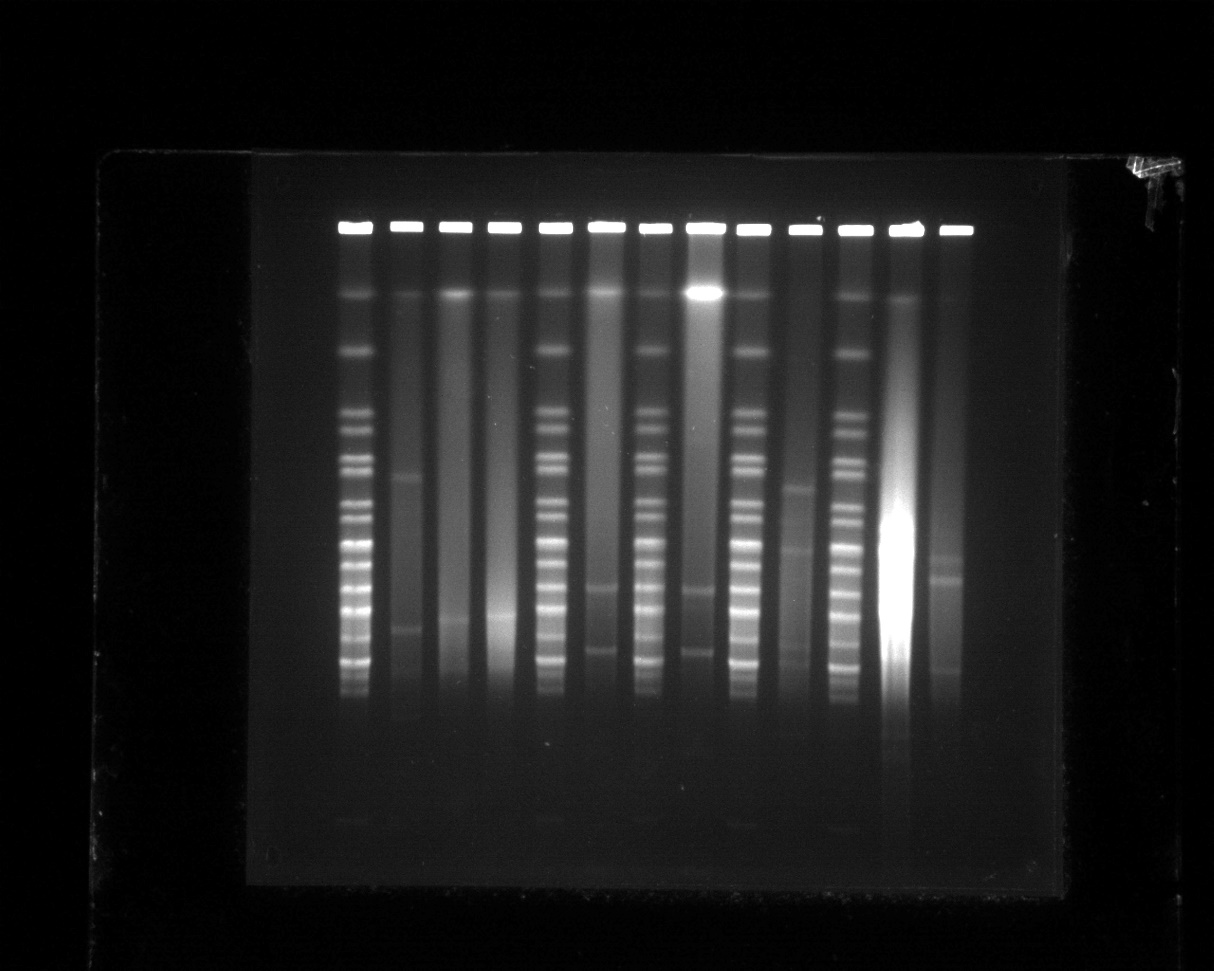


Full-length gel of Fig. 2A. Lane 5-9 was cropped.


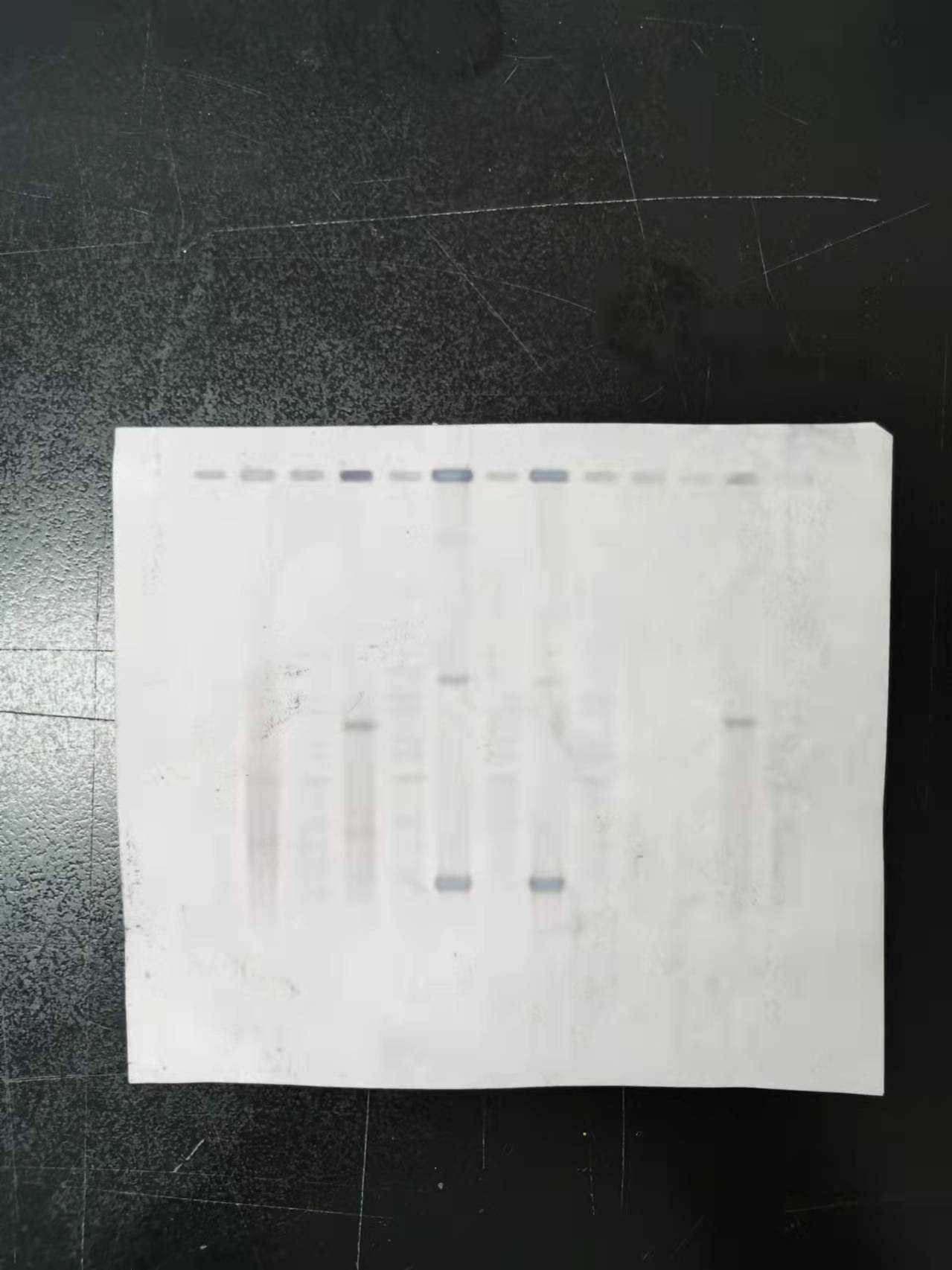


Full-length blot of Fig. 2B. Lane 6-8 was cropped.
