# Supplementary material for: Complete-genome sequencing and comparative genomic characterization of blaNDM-5 carrying Citrobacter freundii isolates from a patient with multiple infections
Source: BMC Genomics. 2023 Aug 30;24:506. doi: 10.1186/s12864-023-09579-9 (PMC10466682; doi:10.1186/s12864-023-09579-9)
Supplement: Supplementary file 2 — Additional file 2: Table S1. Information of DY2007, DY2010 and other 78 NDM-producing C. freundii strains used for phylogenetic tree construction. [file 12864_2023_9579_MOESM2_ESM.docx]

**Table S1** Information of DY2007, DY2010 and other 78 NDM-producing *C. freundii* strains used for phylogenetic tree construction

| **Strain** | **Assembly** | **BioSample** | **Year** | **Location** | **Host** | **ST** |
| --- | --- | --- | --- | --- | --- | --- |
| CRCB-101 | GCA_002786865.1 | SAMN07944242 | 2015 | South Korea: Incheon | Homo sapiens | 427 |
| MH16-522D | GCA_003114935.2 | SAMD00112928 | 2016 | Viet Nam:Hanoi | Homo sapiens | 109 |
| WCHCF65 | GCA_001273815.1 | SAMN03975633 | 2015 | China: Chengdu | hospital sewage | 88 |
| 2021GO-0206 | GCA_021028235.1 | SAMN23521422 | 2021 | USA | Homo sapiens | - |
| F3R | GCA_017151595.1 | SAMN15659132 | 2019 | China: Yangjiang | goose | - |
| strain 146 | GCA_018089965.1 | SAMN05770928 | 2014 | Singapore | Homo sapiens | 534 |
| strain 178 | GCA_016074855.1 | SAMN05770960 | 2014 | Singapore | Homo sapiens | 394 |
| W006P | GCA_016076215.1 | SAMD00108697 | 2017 | Myanmar:Yangon | waste water | - |
| 2018HL-00763 | GCA_015892565.1 | SAMN16980965 | 2018 | USA | Homo sapiens | 328 |
| hkcpe72 | GCA_015893905.1 | SAMN15904741 | 2012 | Hong Kong | Homo sapiens | 129 |
| BSI137 | GCA_016614875.1 | SAMN12109432 | - | - | - | 248 |
| CF_324 | GCA_003937345.2 | SAMN10249196 | 2016 | Pakistan | washroom sink in hospital intensive care unit | - |
| strain 117 | GCA_016074835.1 | SAMN05770899 | 2015 | Singapore | Homo sapiens | 63 |
| 953086287 | GCA_001317155.2 | SAMN04011453 | 2016 | South Africa: Durban | Homo sapiens | 63 |
| ST63:944526466 | GCA_001317135.2 | SAMN04011451 | 2013 | South Africa: Durban | Homo sapiens | 63 |
| C487 | GCA_017901615.1 | SAMD00138032 | 2017 | Thailand:Nakhon Phanom | Homo sapiens | 11 |
| CFM_67 | GCA_011030295.1 | SAMN14120186 | 2018 | Lebanon | Homo sapiens | 91 |
| CFM_69 | GCA_011030315.1 | SAMN14120187 | 2018 | Lebanon | Homo sapiens | 112 |
| 2021GO-0128 | GCA_020321835.1 | SAMN22045168 | 2021 | USA | Homo sapiens | 156 |
| W041V | GCA_016076255.1 | SAMD00108705 | 2017 | Myanmar:Yangon | waste water | - |
| H2730R | GCA_015208815.1 | SAMN12706440 | 2019 | South Africa: Ethekwini district, KwaZulu-Natal | rectal swab | - |
| UCI151 | GCA_014901875.1 | SAMN08148199 | - | USA: CA | Homo sapiens | 18 |
| CF-16-17 | GCA_019659905.1 | SAMN20568206 | 2016 | China | Homo sapiens | 18 |
| C118 | GCA_017535765.1 | SAMD00108862 | 2016 | Thailand: Sakon Nakhon | Homo sapiens | 18 |
| AMA528 | GCA_016088975.1 | SAMEA3712525 | 2013 | Denmark | Homo sapiens | 18 |
| AMA570 | GCA_016089195.1 | SAMEA3712530 | 2014 | Denmark | Homo sapiens | 18 |
| AMA533 | GCA_016088875.1 | SAMEA3712526 | 2013 | Denmark | Homo sapiens | 18 |
| AMA582 | GCA_016089135.1 | SAMEA3712532 | 2013 | Denmark | Homo sapiens | 18 |
| AMA941 | GCA_016089055.1 | SAMEA3712542 | 2015 | Denmark | Homo sapiens | 18 |
| AMA463 | GCA_016089205.1 | SAMEA3712524 | 2013 | Denmark | Homo sapiens | 18 |
| AMA818 | GCA_016089035.1 | SAMEA3712540 | 2014 | Denmark | Homo sapiens | 18 |
| AMA393 | GCA_016089075.1 | SAMEA3712521 | 2013 | Denmark | Homo sapiens | 18 |
| AMA426 | GCA_016088945.1 | SAMEA3712523 | 2013 | Denmark | Homo sapiens | 18 |
| AMA653 | GCA_016089085.1 | SAMEA3712538 | 2014 | Denmark | Homo sapiens | 18 |
| AMA639 | GCA_016089015.1 | SAMEA3712534 | 2014 | Denmark | Homo sapiens | 18 |
| AMA535 | GCA_016089175.1 | SAMEA3712528 | 2013 | Denmark | Homo sapiens | 18 |
| AMA332 | GCA_016089115.1 | SAMEA3712520 | 2012 | Denmark | Homo sapiens | 18 |
| 2020GO-00099 | GCA_017143675.1 | SAMN17767280 | 2020 | USA | Homo sapiens | 261 |
| sc19444294-1 | GCA_018445825.1 | SAMN17371900 | 2019 | Germany | Homo sapiens | 540 |
| microbial | GCA_020920035.1 | SAMN23242575 | 2021 | USA | Homo sapiens | 256 |
| CF-15-33 | GCA_019660585.1 | SAMN20568180 | 2015 | China | Homo sapiens | - |
| CFM_17 | GCA_011030065.1 | SAMN14120105 | 2016 | Lebanon | Homo sapiens | 415 |
| Survcare137 | GCA_018442145.1 | SAMN17372053 | 2018 | Germany | Homo sapiens | - |
| YDC849-1 | GCA_015958925.1 | SAMN14007653 | 2018 | USA: Pittsburgh, Pennsylvania | Homo sapiens | - |
| CB00115 | GCA_016503505.1 | SAMN12220320 | 2018 | USA: Pittsburgh, Pennsylvania | Homo sapiens | - |
| RS259 | GCA_015958745.1 | SAMN14007642 | 2018 | USA: Pittsburgh, Pennsylvania | Homo sapiens | - |
| RS259 | GCA_013336995.1 | SAMN14082846 | 2018 | USA: Pittsburgh, Pennsylvania | Homo sapiens | - |
| 2021DK-00061 | GCA_019270955.1 | SAMN20203196 | 2021 | USA | Homo sapiens | 8 |
| 2021DK-00149 | GCA_020535485.2 | SAMN22329132 | 2021 | USA | Homo sapiens | 8 |
| 2021DK-00063 | GCA_019270935.1 | SAMN20203198 | 2021 | USA | Homo sapiens | 8 |
| W038V | GCA_016076195.1 | SAMD00108702 | 2017 | Myanmar:Yangon | waste water | 340 |
| W054B | GCA_016076095.1 | SAMD00108710 | 2017 | Myanmar:Yangon | waste water | 116 |
| C237 | GCA_017903275.1 | SAMD00137820 | 2016 | Thailand:Surat Thani | Homo sapiens | 116 |
| C238B | GCA_017902215.1 | SAMD00137821 | 2016 | Thailand:Surat Thani | Homo sapiens | 116 |
| TS3W | GCA_017151445.1 | SAMN15659126 | 2018 | China: Yangjiang | soil | 64 |
| Enterobacteriaceae | GCA_021014095.1 | SAMN16679863 | - | China：Shandong | Homo sapiens | 139 |
| CWH001 | GCA_002738435.1 | SAMN07736518 | 2014 | China: Wuhan | Homo sapiens | 139 |
| 119D | GCA_015929735.1 | SAMN15502151 | 2019 | Pakistan | Homo sapiens | 107 |
| 104A | GCA_015929665.1 | SAMN15502146 | 2019 | Pakistan | Homo sapiens | 107 |
| strain 148 | GCA_016075215.1 | SAMN05770930 | 2014 | Singapore | Homo sapiens | 107 |
| strain 147 | GCA_016075155.1 | SAMN05770929 | 2014 | Singapore | Homo sapiens | 107 |
| M607 | GCA_016076295.1 | SAMD00099794 | 2016 | Myanmar:Yangon | Homo sapiens | 22 |
| microbial | GCA_019942435.1 | SAMN21370287 | 2021 | USA | Homo sapiens | 22 |
| EPINDM23 | GCA_019061035.1 | SAMN19729922 | 2016 | France: Paris | Homo sapiens | 22 |
| EPINDM61 | GCA_019060165.1 | SAMN19729923 | 2016 | France: Paris | Homo sapiens | 22 |
| AUSMDU00005023 | GCA_016081375.1 | SAMN12342906 | 2015 | Australia | Homo sapiens | 22 |
| MGF016 | GCA_004795555.1 | SAMN11366429 | 2015 | Malaysia: Johor | Homo sapiens | 22 |
| Citrobacter_freundii_2021CK-01033 | GCA_018868325.1 | SAMN19677621 | 2021 | USA | Homo sapiens | 22 |
| 149j4 | GCA_014803225.1 | SAMN16205289 | 2017 | France | Homo sapiens | 22 |
| M625 | GCA_016076155.1 | SAMD00129399 | 2016 | Myanmar:Yangon | Homo sapiens | 22 |
| DY2010 | GCA_020809005.1 | SAMN21601121 | 2020 | China: Dongyang | Homo sapiens | 22 |
| DY2007 | GCA_020639355.1 | SAMN21601015 | 2020 | China: Dongyang | Homo sapiens | 22 |
| Survcare429 | GCA_018421175.1 | SAMN17373092 | 2019 | Germany | Homo sapiens | 22 |
| CF-16-61 | GCA_019660345.1 | SAMN20568216 | 2016 | China | Homo sapiens | 22 |
| strain 151 | GCA_016075055.1 | SAMN05770933 | 2014 | Singapore | Homo sapiens | 22 |
| strain 149 | GCA_016075095.1 | SAMN05770931 | 2014 | Singapore | Homo sapiens | 22 |
| strain 152 | GCA_016074955.1 | SAMN05770934 | 2014 | Singapore | Homo sapiens | 22 |
| CF-14-50 | GCA_019660945.1 | SAMN20568165 | 2014 | China | Homo sapiens | 22 |
| CF-15-61 | GCA_019660505.1 | SAMN20568179 | 2015 | China | Homo sapiens | 22 |
| CF-15-2-29 | GCA_019660135.1 | SAMN20568193 | 2015 | China | Homo sapiens | 22 |
