# Supplementary material for: Complete-genome sequencing and comparative genomic characterization of blaNDM-5 carrying Citrobacter freundii isolates from a patient with multiple infections
Source: BMC Genomics. 2023 Aug 30;24:506. doi: 10.1186/s12864-023-09579-9 (PMC10466682; doi:10.1186/s12864-023-09579-9)
Supplement: Supplementary file 3 — Additional file 3: Table S2. List of information for both genomes that were sequenced in this study. [file 12864_2023_9579_MOESM3_ESM.docx]

**Table S2** List of information for both genomes that were sequenced in this study

| **Strain** | **Genome size (bp)** | **N50 value** | **L50 value** | **GC content (%)** | **Number of contigs** | **Plasmid info** | **ST type** | **Antimicrobial resistance genes** |
| --- | --- | --- | --- | --- | --- | --- | --- | --- |
| DY2007 | 5,253,532 | 4932911 | 1 | 51.6 | 4 | IncX3 | ST22 | *bla*_TEM-1B_, *bla*_OXA-1_, *bla*_CMY-48_, *bla*_DHA-1_, *aac(6')-Ib-cr*, *aac(3)-IId*, *aadA1*, *qnrB4*, *tet(D)*, *mph(A)*, *dfrA1*, *sul1*, *sul2*, *catA2*, *catB3*, *ARR-3* |
| DY2010 | 5,260,876 | 5092352 | 1 | 51.6 | 4 | IncX3 | ST22 | *bla*_TEM-1B_, *bla*_OXA-1_, *bla*_CMY-48_, *bla*_DHA-1_, *aac(6')-Ib-cr*, *aac(3)-IId*, *aadA1*, *qnrB4*, *tet(D)*, *mph(A)*, *dfrA1*, *sul1*, *sul2*, *catA2*, *catB3*, *ARR-3* |
